# Sulphæmoglobinæmia

**Published:** 1911-05-27

**Authors:** 


### Medicine.

### SULPHÆMOGLOBINÆMIA.

This somewhat formidable title has been given to a very puzzling blood condition revealed by spectroscopic hæmatology in recent years. Some five years ago Van der Bergh, of Rotterdam, was investigating a case of persistent cyanosis in a boy suffering from rectal stricture. He at first believed that he was dealing with a case of methæmoglobinæmia, a condition first described by Stokvis; but eventually it was proved that the abnormal blood pigment was sulphæmoglobin, a purple pigment which was produced many years ago *in vitro* by Claude Bernard by the action of sulphuretted hydrogen upon blood. The following year Van der Bergh reported three more cases; in this series of four, cure resulted in each case after systematic purgation, and the discoverer was thus led to conclude that the abnormality is due to absorption of sulphuretted hydrogen from the bowel. In England the first reported case was observed in 1906 by Drs. West, Garrod, and Clarke at St. Bartholomew's Hospital; a report was made to the Royal Medico-Chirurgical Society the following year. In this instance a searching investigation failed to show whence the blood derived its unusual constituent. The only suggestion that commended itself was that owing to some unrevealed pathological condition the blood may have been enabled to combine chemically with traces of sulphuretted hydrogen normally dissolved in the plasma. This, however, is a purely hypothetical view of the ætiology of the condition, unsupported by clinical findings. Within a short time of the publication of this case, two more similar cases were reported in London by Drs. Russell and Wynter respectively. Since then the condition has been identified in America, where Drs. Clarke and Curtis have analysed it recently for the *Medical Record*, and have reported a case.

The clinical characteristics of this peculiar lesion are few, but striking. Without any obvious reason in some cases, or after continued constipation in others, the patient's skin assumes a tint which is described as cadaveric, or better as "the ghastly hue seen in a person standing under the Cooper-Hewitt mercury light." The lips are deep purple, almost black, and the skin is of a steel-blue tinge. There is no pain: indeed subjective symptoms may be entirely lacking, and the patients themselves feel well, although their relatives believe that death must be imminent. Physical examination reveals nothing abnormal except this coloration which affects the whole body. The diagnosis can only be made by the spectroscopic examination of a few drops of blood from the ear. This is easily done, and does not require any very complicated apparatus. The blood is of a rich chocolate-colour when withdrawn; it is mixed with twice its volume of distilled water before clotting takes place and thoroughly shaken. After the fibrin has separated the solution is filtered several times through one filter paper. The clear solution is looked at through a simple inexpensive spectroscope; and is then diluted drop by drop with water until the red colour stands out clearly. If there is a black absorption band in the red end of the spectrum, either methæmoglobin or sulphæmoglobin is present; if this band persists after adding a drop of ammonium sulphide solution, the pigment is sulphæmoglobin, but if it disappears, methæmoglobin.

Both these blood diseases have a good ultimate prognosis as far as life goes, though recovery may take a very long time. Since the aspect of the patient suggests immediate dissolution, it is important to recognise the condition, and to do whatever is necessary in the way of treatment. Methæmoglobinæmia occurs chiefly in the male sex; sulphæmoglobinæmia chiefly, but not entirely in the female. For the latter condition no special diet has been found to be of any great use, though many kinds have been experimented with; even an all-milk *régime*, which temporarily banishes methæmoglobin from the blood, has no influence over sulphæmoglobin. The regulation of the bowels is of the utmost importance, and patients in whom this has been successfully accomplished in hospital, with cure of their cyanosis, have relapsed again speedily on returning home and once more becoming subject to constipation. Apart from regular purgation, rest in bed with massage and intestinal antiseptics seem to give the best results. Of the latter, some success has been obtained with salol and -naphthol. The ætiology of the disease is at present so indefinite that any line of treatment must be as yet somewhat empirical. More reported cases thoroughly investigated are needed before the condition can be subjected to a study sufficiently searching to yield a knowledge of it more intimate than that which we now have. There is little doubt that there are a good number of unrecognised cases; most of them are diagnosed as congenital morbus cordis, and regarded as incurable.

